# Optimization and Security of Hazardous Waste Incineration Plants with the Use of a Heuristic Algorithm

**DOI:** 10.3390/s21217247

**Published:** 2021-10-30

**Authors:** Agata Wajda, Tomasz Jaworski

**Affiliations:** Department of Technologies and Installations for Waste Management, Faculty of Energy and Environmental Engineering, Silesian University of Technology, Konarskiego 18, 44-100 Gliwice, Poland; Tomasz.Jaworski@polsl.pl

**Keywords:** process security, rotary kiln, optimization application, ant colony algorithm, hazardous waste, waste incineration plant

## Abstract

The amount of generated waste, which increases every year, is a serious problem of the modern world. In particular, attention should be paid to hazardous waste and methods of its disposal. One of the most used in this context is thermal treatment in dedicated incinerators equipped with a rotary kiln. Conducting the process requires, inter alia, supplying the furnace with a batch of batch material with appropriate parameters. Improper operation in this regard may cause negative environmental effects and operational problems. The key here is to select different types of hazardous waste and compose batch portions. The paper presents an application that optimizes the work of waste incineration plant operators. At the same time, this tool can be described as ensuring security at this stage of the process. The application implements an ant colony algorithm that selects the optimal solution to the problem, which has been formulated here as the types and masses of the batch mixture components with given parameters. The application has been tested in the laboratory and real conditions with satisfactory results.

## 1. Introduction

Rotary kilns are widely used in the cement, chemical, metallurgical, and waste management industries. The structure of the furnace can be described as an elongated, cylindrical steel drum with a certain diameter in an almost horizontal position. As the device works most often at high temperatures, the interior of the furnace is made of fireproof lining. The furnace rotates around its axis with a rotational speed in the range of 0.25–4.5 revolutions/minute. In the case of thermal processing of waste, the rotary kiln most often carries out the incineration of hazardous waste [1,2,3,4,5].

An important area for optimizing the operation of a rotary kiln hazardous waste incineration plant is the proper establishment of the feed material composition. This selection is of great importance for the operation of the facility. It should be emphasized that there is a negligible amount of specialist literature describing the influence of the properties of the charge material on the operation of a rotary kiln in a hazardous waste incineration plant. As part of the literature review, one publication [6] can be cited, in which the authors examine the use of lining bricks. They point out the need to regulate the basicity of the bottom ash in order to avoid its high alkalinity, as it favors the infiltration of the lining.

In the works of other authors, an approach indicating thermochemical corrosion is visible as a phenomenon that affects the lining to the greatest extent. The publications referenced herein relate to a rotary cement kiln or a kiln for the firing of metals. The processes taking place in them are characterized by different parameters, however, it is worth quoting some theses of these works, as they are also justified in the case of rotary kilns in incinerators. The author [7] emphasizes that alkaline corrosion takes place at higher temperatures, leading to the degradation of the lining. The research carried out by [8] presents the view concerning the dependence of the operating conditions of the furnace on the thickness of the agglomerated material in the furnace. While the thin layer protects the lining, excessive formation of alkaline deposits can deteriorate the flow of material through the furnace and promote brick degradation. The attention to alkaline salts as the main cause of lining destruction was also undertaken in the works [9,10]. The authors of the study [11] aimed to determine the effect of incineration of municipal waste in a cement rotary kiln on the lining. The result indicates that corrosion is the most important degradation mechanism for bricks. In chemical terms, they distinguished the reactions between the gases emitted during the thermal process containing alkali, sulfur, and chlorine, and the refractory material, as a result of which chloride, sulphate, and complex salts are formed. They are characterized by a low melting point and the ability to metamorphize the lining material, which takes a loose form, prone to chipping.

Nevertheless, none of the cited references has developed a tool supporting the work of operators and did not refer to the aspect of proper preparation of the waste mixture fed to the furnace. In some papers, for example in [5], general guidelines for the implementation of the process can be found, such as avoiding highly alkaline wastes, regular monitoring of the alkalinity of the ashes in the kiln, recommending the use of lower process temperatures for alkaline wastes. Therefore, in order to develop a supporting tool, information obtained from the operators of these installations was used, their experiences, and possible procedures (if any) were used.

Hazardous waste is often characterized by properties that significantly exceed those of standard fuels, as well as waste fuels, such as a high content of halides or alkaline salts. Hence, the batch material for the furnace is often a composition of several types of hazardous waste, so that their specific features are mutually exclusive and do not affect the thermal process. These parameters are most often determined to some extent by the incineration laboratory, while at a later stage the selection of the mixture fed to the furnace for incineration depends on the operator. The operator’s action in this regard is based on numerous observations and experience. This requires a lot of time and may be subject to certain unfavorable events, which in the most glaring cases may be associated with the shutdown of the installation, temporary or longer.

Bearing in mind the above, a desktop application was developed to support the work of users of waste incineration plants. The purpose of this application is to enable the operator of the incineration plant to automatically select the components of the mixture, which is a portion of the input material for the furnace. It is suitable for Windows computers. It was written in NET technology in the C# programming language based on the ant colony algorithm.

## 2. Materials and Methods

Appropriate selection of the portion of the supplied batch material is a painstaking work that requires knowledge, experience, and efficient cooperation of individual departments of the waste incineration plant. Nevertheless, even assuming that all conditions are met, problems may arise that affect the operation of the entire installation. They are correlated with the properties of some types of waste, which have a large negative impact on the installation. When carrying out the thermal process, it is important to keep the input material at a uniform level as far as possible in terms of physicochemical and fuel properties. In addition, these properties must meet the appropriate requirements, which allows the process to be conducted in accordance with the legislation imposed. It is not easy when it is necessary to transform various types of waste, such as waste from alkaline purification of fuels with a high content of alkaline salts, high pH, and low calorific value, acid tars with a high sulfur content, low pH and caloric value, or high calorific oil filters and many others. The number of waste parameters taken into account is also considerable, although here it may be generally limited to the fuel properties and some specificities that can result in uncontrolled processes in the kiln.

The consequences of improper selection of the batch material portion may take various forms, from a short-term peak in the emission of a given gas, through a temporary stagnation of material in the furnace, to the necessity to stop the installation and perform renovation due to, for example, wear of the kiln lining. This is directly related to ensuring the security of thermal waste treatment installations.

### 2.1. Assumptions of the Application Model

**Figure 1:**
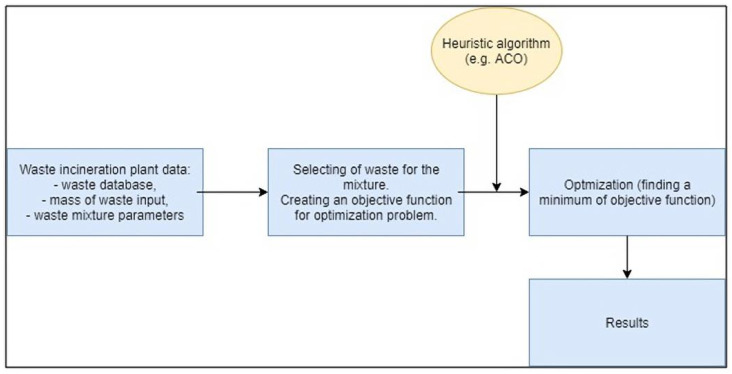
General scheme of the application model.

The application provides for the selection of waste types, previously selected from the list, in a way that allows the creation of a waste mixture with preset values of output parameters. The parameters have been defined with regard to their usefulness in the assessment of a given material for the combustion process, its impact on the environment and operational aspects. Then, each of the parameters was assigned a range of values appropriate for the implementation of the thermal process, and going further from the ranges of individual parameters, reference values were selected, being the desired value of a given parameter. The general scheme of the application model is presented in Figure 1.

Based on the data obtained from operators of thermal treatment installations for hazardous waste, the following parameters were selected: calorific value, pH, chlorine content, basic salt content (Na, K, Ca), and halide content (F, I, Br).

Due to the fact that no data on the operating parameters of the rotary kiln in the incinerator were found in the literature, the optimal conditions, here ranges, for the thermal process were determined on the same basis. In the next step, model values in the range were determined for each parameter. Value ranges for a given parameter are based on information and experiences from real-world objects. The list of parameters and reference values is presented in Table 1.

These values are determined experimentally and are primarily aimed at the correct conduct of the thermal process in the furnace in terms of operation, which translates into the financial aspect. Namely, maintaining an autothermal process with a relatively uniform temperature distribution can be achieved by using a charge with a fixed calorific value. As a result, it is not necessary to feed additional fuel to the process in the rotary kiln. On the other hand, acidic or alkaline substances in too high concentrations, which persist for a long time, cause faster wear of the lining. This is related to its more frequent renovation, which is an undesirable cost. In addition, the incinerator does not carry out a thermal process at this time and therefore does not generate revenues.

In the case of calorific value and pH, the standard value was selected as the average value, while for parameters with a limit value, it was indicated as a standard value constituting 10% of the maximum value set. The selection of the reference value has an additional advantage, which is the model’s resistance to certain deviations in the operation of the algorithm indicating the output values —there is a relatively large margin of error, at the same time allowing to obtain output values in accordance with the specified ranges of parameters.

Another important assumption concerns the initial values of the individual parameters of the mixture. They are assumed to be the weighted averages of its components, where weight is the weight of the component in the blend.

### 2.2. Algorithm for Selection of Components of the Input Material

In the first step, an objective function was created which was defined as follows:

(1) $$f\left(s_{1},\;s_{2},\ldots ,\;s_{n}\;\right)=\sum_{i=1}^{5}\left({\bar{p}}_{i}-p_{wi}\right)x^{2}$$

where $s_{1},\;s_{2},\ldots ,\;s_{n\;}—$ masses (weights) of individual components, *n*—number of ingredients, ${\bar{p}}_{i}$—weighted average of the parameter with index *i*, $p_{wi}$—reference value of the parameter.

It is a function of n variables, where n is the number of components that can be in the mixture. It is then minimized relative to the weight of the ingredients, where weight is their weight. The objective function describes the quality of the mixture in relation to certain parameters. The smaller the value of this function, the better the parameters of the mixture, in this case, closest to the reference values will be.

One of the artificial intelligence algorithms, belonging to the group of swarming algorithms, was used to minimize the objective function—the ant algorithm, described in more detail below. This algorithm is characterized by a relatively simple implementation. Moreover, it is not necessary to make assumptions about the nature of the objective function, namely its continuity or differentiability. The operation of the algorithm can be simplified as follows: the algorithm receives a set of waste from which a mixture is to be made, and then selects the masses of individually selected waste so that their mixture is characterized by model values [12,13,14,15,16].

The ant algorithm is based on the behavior of an ant colony in nature when searching for food. These creatures communicate through pheromone tracks left in the way, indicating the path they travel. They take their route often in the most optimal way, i.e., in this case these are short routes that allow you to obtain food and return to the anthill. The pheromone trail on these routes is stronger, causing subsequent ants to choose this route over time, which intensifies the intensity of the pheromone trail. This observation of nature, written in mathematical form, allows it to be adapted to solve many optimization problems [13].

The goal of naturally occurring ants is to efficiently reach the food site, which in the algorithm means finding the minimum of the target function. This mechanism can be described as follows. In general, you should start with determining the algorithm parameters, such as the number of ants *M*, the number of iterations *I*, and the vector of the initial jump parameters *β*^1^ = [*β*_1_^1^, *β*_2_^1^, …, *β_n_*^1^], where n is the dimension of the task or the number of variables. The solutions (vectors *x^k^*, *k* = 1, 2, …, *M*) are ants in the algorithm. Initially, they are placed randomly. In each iteration of the algorithm, the solution with the best characteristics is determined—*x^best^*, which, using a reference to nature, denotes the ant closest to the food. In the next step, the vector of offsets *dx* = (*dx*_1_*, dx*_2_, …, *dx_n_*) is added to this solution. This vector is created on the basis of the algorithm parameters *β_j_^i^* (*j* = 1, 2, …, *n*, *i* = 1, 2, …, *I*), where *dx_j_* є [−*β_j_^i^, β_j_^i^*]. The task of *β_j_^i^* parameters is to narrow the area of searching for a solution around the most optimal solution. The initial vector of the parameters *β*^1^ at the beginning of the algorithm should be set here. In subsequent iterations, these parameters will be determined from the following dependency *β_j_^i^*^+1^ *=* *ξ ·* *β_j_^i^,* where *ξ* є [0, 1] and it is the parameter narrowing the search area. There is a relationship here: the smaller the value of the parameter *ξ*, the more narrowed the search area. This is how new ant distributions are created, which will be updated repeatedly in *I*^2^ in each iteration of the process. For each iteration of the algorithm, except the first, the parameter *β^i^* is changed. Such action is to simulate the disappearance of the pheromone trace.

The presented approach is based on the algorithm described in [15]. The algorithm has been adapted to parallel computations for the needs of application development. The following markings were introduced in it:

*F*—minimized function (objective function),

*n*—task dimension,

*D*—task area, *x* = (*x*_1_, *x*_2_, …, *x_n_*) є *D nT*—number of threads,

*M = nT p*—number of ants in the population,

*I*—number of iterations,

*β^i^*—vector of jump parameters,

*ξ*—narrowing parameter.

**Figure 2:**
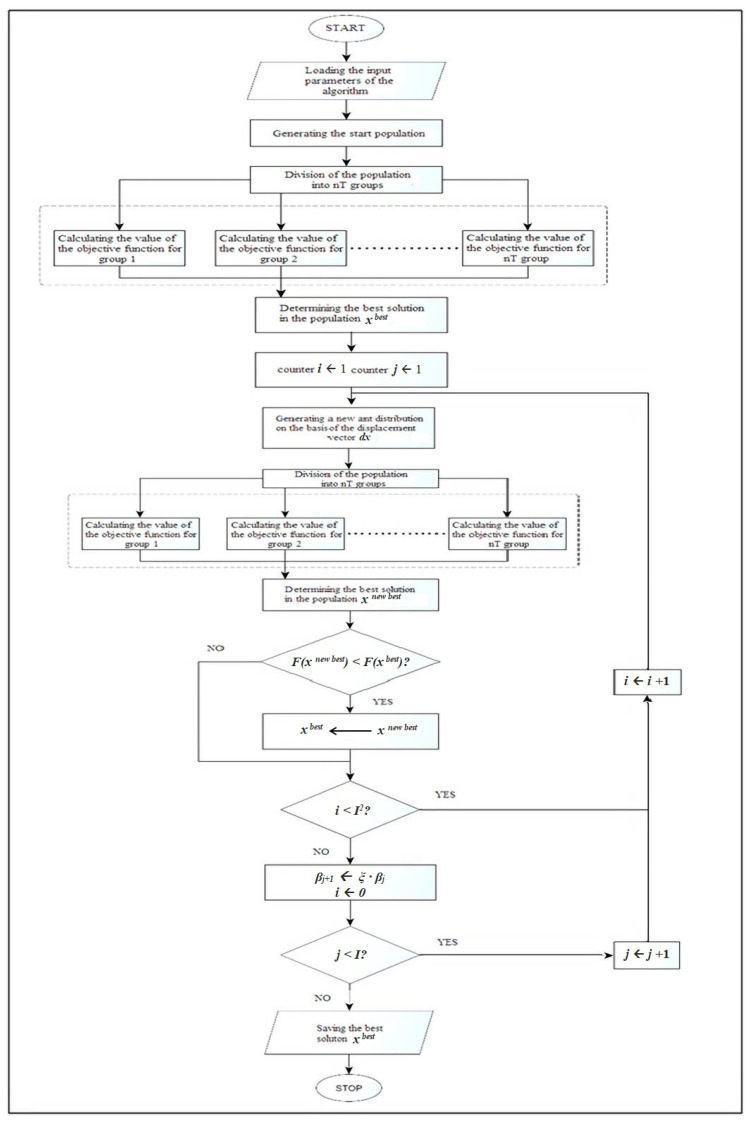
Scheme of the ant colony algorithm.

Figure 2 shows scheme of the adopted ant colony optimization algorithm.

Operation with the use of an algorithm can be divided into two stages: start and iterative.

The following actions take place during the start of the algorithm:

- Setting the input parameters of the algorithm: *M, I, nT, ξ, β*^1^.
- Generating the starting population *x^k^ = (x*_1_*^k^*, *x*_2_*^k^*, …, *x_n_^k^),* where *x^k^* є *D*, *(k =* 1, 2, …, *M).*
- Breakdown of the population into *nT* groups that will be combined in parallel.
- Finding the value of the objective function for each ant in the population for parallel computations.
- Determination of the best *x^best^* solution in the population.

Within the iterative stage, the following steps are defined:

- Random generation of the shift vector *dx^k^* = *(dx*_1_*^k^*, *dx*_2_*^k^*, …, *dx_n_^k^*), where *–β_j_^i^* *≤ dx_j_^k^* ≤ *β_j_^i^ (k* = 1, 2, …, *M*).
- Generation of a new distribution of an ant colony *x^k^* = *x^best^* + *dx^k^*, (*k* = 1, 2, …, *M*).
- Division of the new colony into *nT* groups counted in parallel.
- Determining the value of the objective function for a new ant colony—parallel calculations.
- Determining the best solution in the current ant colony. In case of a better solution than *x^best^*, it is assumed as a new solution *x^best^.*
- Steps 6 to 10 are repeated *I* times.
- Decreasing the values of the parameters *β_j_^i^*^+1^*: β_j_^i^*^+1^ *= ξ · β_j_^i^.*
- Steps 6 to 12 are repeated *I* times.

Ant algorithm parameters.

If the mixture is to contain less than six different types of waste, then the following parameters are taken into account:

- number of ants *M* = 30;
- number of pheromone spots *L* = 10
- number of iterations *I* = 30

As a result of the following selection of parameters, the number of calls to the objective function will be 910. In the case of more types of waste, the number of ants and pheromone spots is increased twice. The authors’ experience shows that the adoption of the following parameters of the ant algorithm makes the algorithm work stable and the obtained results are satisfactory.

**Figure 3:**
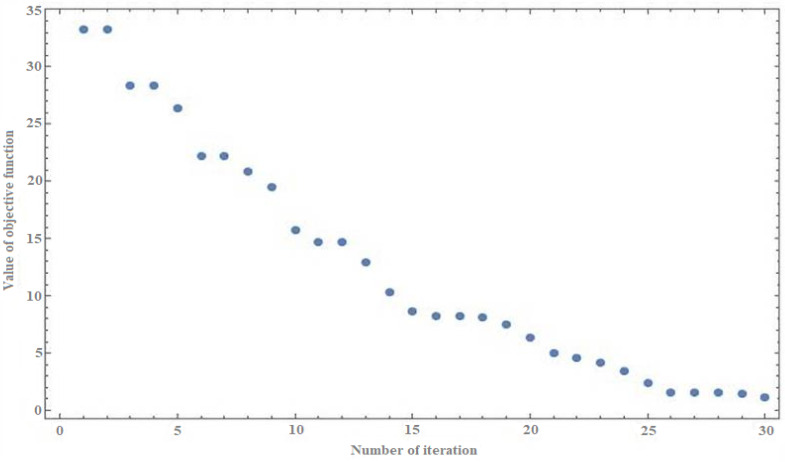
Values of the objective function in subsequent iterations of the algorithm.

The results can be considered satisfactory due to two criteria. The first is the fact that the values of certain parameters of the obtained waste mixture are close to the reference values and are within the given value ranges. The second criterion is the evaluation of the stability of the algorithm—for the generated solution, the value of the objective function should be relatively close to zero. In this approach, these values are checked in subsequent iterations of the algorithm, as shown in Figure 3.

As can be seen in Figure 3, the value of the objective function for the solution is relatively close to zero. This example simulates the formation of a batch material mixture from 15 selected waste types for a given batch mass of 150 kg. The stability of the solution can also be noticed here in the case of the task dimension (the number of ingredients in the mixture) with more than 10.

## 3. Results

The developed application of the selection of waste mixture components is aimed at an optimal composition of the batch material portion for the rotary kiln in the hazardous waste incineration plant. Optimal means ensuring the maintenance of specific parameters of the installation and minimizing operational problems occurring in the incineration plant.

The application takes into account five selected parameters, i.e., calorific value, pH, chlorine content, alkaline salt content, and the content of other halogens. Each parameter is assigned a range of values within which the obtained results should be included.

The assumption that guided the development of the application was the ease of use, flexibility, stability of solutions, and the possibility of direct use in the work of a waste incineration plant operator. The results here can be described as the results of application tests in terms of meeting the functions important from the point of view of the operator of the installation. They are:

- Creating a mixture that meets the given parameters;
- Compatibility with the database of waste incineration plant;
- Selecting waste for a batch of input material;
- Updating resources after selection;
- Generating reports.

It is worth noting here that the specific functions of the application resulting from its intended use are in line with the basic assumption of the application, which is ensuring the security of hazardous waste incineration plants in terms of the impact on the environment and impact on people, as well as an attempt to optimize its operation.

### 3.1. Creating a Mixture That Meets the Given Parameters

**Figure 4:**
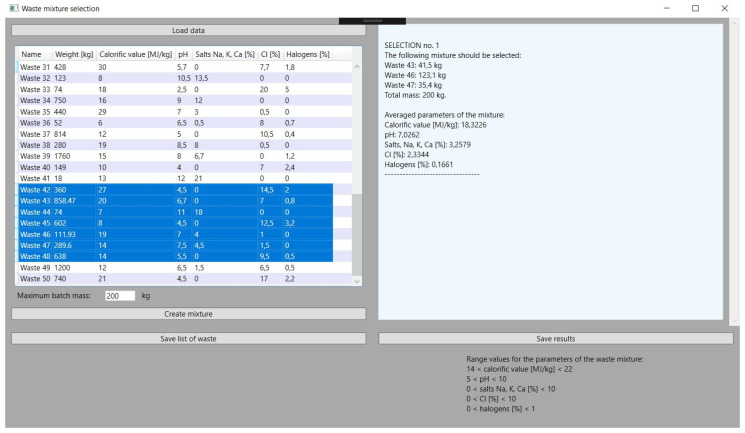
Results of the first received batch mixture.

This is a key feature of the application on which its development was based. The objective function, described in the previous chapter, gives very satisfactory results thanks to a properly selected problem-solving algorithm. In all the tests performed, the values of the parameters of the obtained mixture are close to the set reference values, which are within the permissible, assumed ranges of the selected parameters of the waste with a large margin of error. Hence, the obtained results are characterized by high stability and are consistent with the assumed guidelines. Exemplary application tests, i.e., in this approach, checking the obtained parameters of the mixture, the mass of its components, and the specified sum of the total batch of the batch material, can be compared with the ranges of parameter values in Figure 4.

### 3.2. Compatibility with the Database of Waste Incineration Plant

In the application, it is possible to load data directly from another file with a specific format. Here, the Excel file was selected due to its widespread use. Thanks to this function, it is possible to directly download data from files generated by the laboratory after analyzing the properties of the received hazardous waste. This is one of the elements of making the application easy to use. There is no upper or lower limit to the number of wastes that can be loaded, although it must be borne in mind that you need to load at least two for a mixture to form.

### 3.3. Selecting Waste for a Batch of Input Material

**Figure 5:**
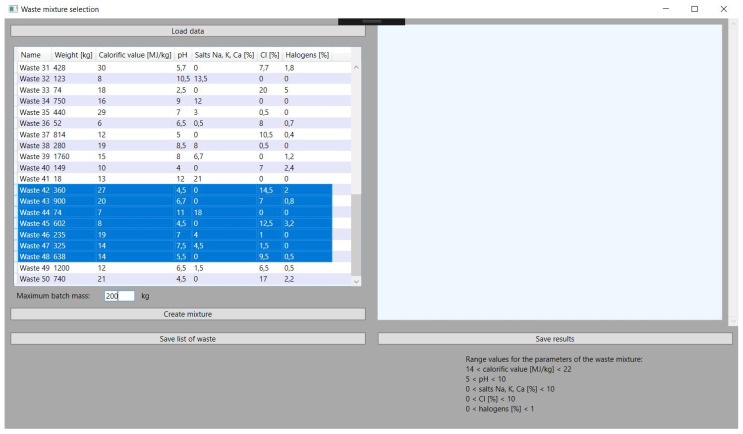
Selection types of waste from downloaded database of incineration plant.

The function of selecting specific waste for the mixture from among all those loaded in the application is an element that makes it flexible. The application user must select the types of waste before obtaining the data about the mixture, but he can do it in many ways depending on the needs. He can choose several types of waste that he wants to convert the most. In hazardous waste incineration plants, there are sometimes situations in which some of the received waste cannot be stored or can, but to a very limited extent, be stored. This is due to the nuisance of these wastes, and hence there is a certain prioritization in directing these wastes to incineration plants. Often, such wastes will require the selection of other wastes with opposite parameters to the batch mixture, hence the solution enabling the selection of several is adequate here. It should be mentioned here that the selection of a certain amount of waste is not tantamount to using each of them in the generated mixture. On the other hand, it is also possible to select considerably more waste until all are selected. This gives the plant operator a lot of room for maneuver. Selected types of waste are marked in blue in the application, as shown in Figure 5.

### 3.4. Updating Resources after Selection

**Figure 6:**
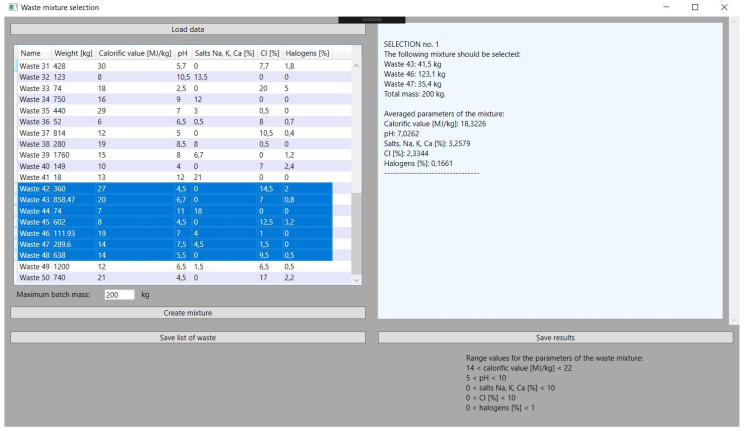
Selection of types of waste to the input waste material mixture.

An important issue that has been programmed is the updating of the mass of waste used to compose the mixture. This function is necessary for the continuous operation of the incinerator. It is about the possibility of creating further blends. The loss of mass can be checked by comparing Figure 5, which shows the selection of waste for the mixture before starting the selection of waste, with Figure 6, which shows the results obtained. The application automatically subtracted appropriate masses from the waste used for the mixture as soon as the results were indicated.

**Figure 7:**
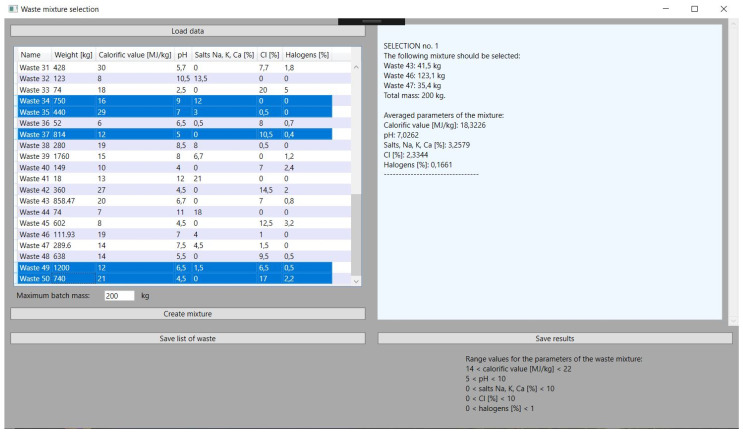
Selection of the next mixture from other types of waste.

**Figure 8:**
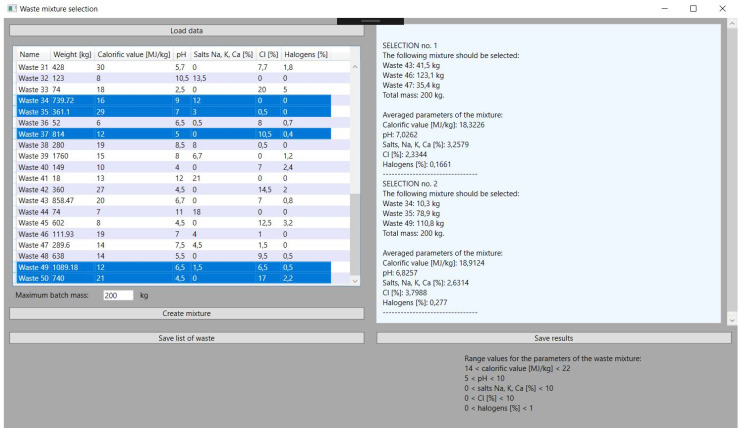
Results obtained for the second mixture.

**Figure 9:**
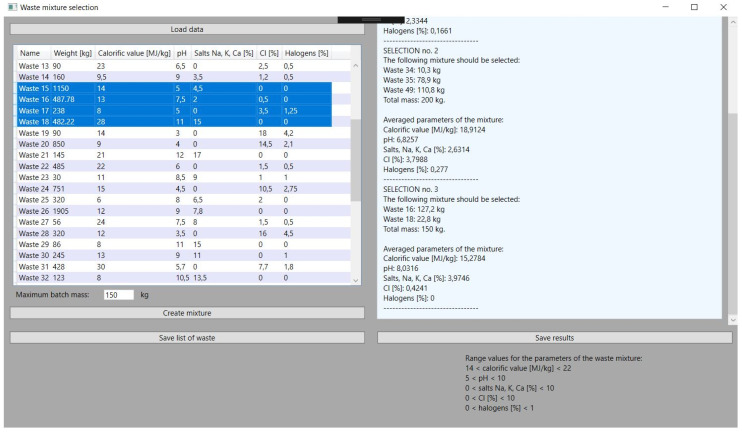
The change in the mass mixture.

Due to the fact that the waste incineration plant is loaded with high frequency during the day, updating the waste mass and creating many mixtures of waste is necessary. The application has no upper limits set in this range. The limiting factor, however, may be the total use of waste. The tests also in the case of simulating multiple selections of waste to the mixture proved to be stable and met the set parameters. This can be seen in Figure 7, Figure 8 and Figure 9, which illustrate successive attempts to select a mixture of waste. In each of the results obtained, hereinafter referred to as steps, the simulated batch of feed material meets the requirements.

### 3.5. Generating Reports

The last function, but extremely important from the plant operator’s point of view, is the ability to save the generated data for individual waste mixtures and create a report on this basis. This function is intended to facilitate the management of loading. On the basis of all these functions, the user of the application can obtain a report containing guidelines for the portion of the input material in a given time frame. Knowing the frequency of loading in a given installation, it is possible to obtain loading schedules designated by the application for batch material portions for one day or week. Wider ranges due to the dynamics of waste incineration are unlikely to be relevant here but are also possible to demonstrate. Figure 9 shows the formation of input materials portions with a changed mass of the portion material.

## 4. Discussion

The developed application of the selection of the components of the batch material in a hazardous waste incineration plant is a tool that has not been found so far neither in the scientific literature nor in commercial product catalogs. It was created on the basis of information obtained from operators of this type of installation and a certain type of need to improve their work. An important aspect here is also the aspect of minimizing operational problems that may occur with the wrong selection of a charge portion of hazardous waste.

The created tool is simplified and requires some further improvements to increase the freedom of use. An open question is also the method of determining the mass composition of the mixture on the basis of the weighted average (resulting from the mass of the component). It does not assume specific chemical reactions occurring at high temperatures between compounds. Nevertheless, it is an attempt to facilitate the operation of the incinerator and is based on the daily work of the operators of these installations, who also do not test the effects of individual substances on each other. Moreover, the subject of research on this issue was not found in the literature. This is a practical aspect of rotary kiln operation that is extremely difficult to define.

The proposed solution assumes, among other things, the acceleration of the decision-making process and the creation of a loading schedule, as well as minimization of operational problems. Application tests, both in the laboratory and real conditions, gave satisfactory results in the context of selected application functions. In addition to fulfilling the basic task, i.e., creating a balanced mixture of input material, the operators of the installation in which the initial tests were carried out positively enhanced the possibility of generating reports. This is important to them for two reasons:

- Automatic creation of an input portion, which reduces the workload.
- Data archiving enabling the comparison of individual types of hazardous waste with generated emissions of exhaust gases.

The second reason is an introduction to consider the possibility of a significant expansion of the application, practically into a system that would combine the selection of batch components with the ongoing monitoring of gas emissions carried out in hazardous waste incineration plants.

## 5. Conclusions

The safety of waste incineration plants, especially hazardous waste, is an important issue from the point of view of environmental protection and possible negative impact on human health. The economic aspect important for the management of thermal waste treatment installations is also important. Any operational errors may result in costly repairs or higher fees for using the environment. Hence, the idea of developing a tool for selecting the components of the mixture of the batch material. The proposed solution has not been so far recognized in the literature. Due to the practical approach to the problem to be solved, cooperation with a hazardous waste incineration plant was established.

The algorithm used in the application is based on the objective function, which determines the method of selecting the mixture components and the generated results. The selected ant algorithm and the parameters matched to it proved successful in solving the problem under consideration. In the tested examples, the obtained waste portions were characterized by parameters within the set value ranges, regardless of the number of wastes in the mixture. This indicates the stability of the proposed approach.

Future directions of application development activities can be considered in several aspects. One of them is further cooperation with representatives of the real object. Despite the extensive experience of the incinerator operators, they are interested in further testing of the developed tool. In practical terms, the application will be developed in such a way as to increase compatibility with systems used in the incineration plant. It is also planned to try to extend the functionality of the application. Another aspect of the work on the application is the development of a detailed analysis of the selection of the parameters of the ant algorithm, as well as the comparison of the ant algorithm with other heuristic algorithms.

## Figures and Tables

**Table 1:** Assumptions of the application parameters and their values.

Parameter	Symbol	Unit	Value Range	Reference Value
Calorific value of waste	P1	MJ/kg	14–22	18
pH	P2	-	5–10	7.5
Content of Cl	P3	% mass	<10	1
Content of salts Na, K, Ca	P4	% mass	<10	1
Content of F, I, Br	P5	% mass	<1	0.1

## Data Availability

Not applicable.

## References

[1] Lemieux P., Boe T., Tschursin A., Denison M.K., Davis K. (2021). Computational simulation of incineration of chemically and biologically contaminated wastes. J. Air Waste Manag. Assoc..

[2] Bujak J., Sitarz P., Nakielska M. (2021). Multidimensional analysis of meat and bone meal (Mbm) incineration process. Energies.

[3] Zhao H.-L., Wang L., Liu F., Liu H.-Q., Zhang N., Zhu Y.-W. (2021). Energy, environment and economy assessment of medical waste disposal technologies in China. Sci. Total Environ..

[4] Wajda A., Jaworski T. (2019). Research on the incineration process of the solid waste in a rotary kiln. Int. Multidiscip. Sci. GeoConference Surv. Geol. Min. Ecol. Manag..

[5] Gajendra K., Shabina K. (2017). Computational fluid dynamics of sponge iron rotary kiln. Case Stud. Therm. Eng..

[6] Meyer V., Pisch A., Penttila K., Koukkari P. (2016). Computation of steady state termochemistry in rotary kilns: Application to the cement clinker manufacturing process. Chem. Eng. Res. Des..

[7] Njeng A., Vitu S., Clausse M., Dirion J.-L., Debacq M. (2018). Wall-to-solid heat transfer coefficient in flighred rotary kilns: Experimental determination and modelling. Exper. Therm. Fluid Sci..

[8] Martha T.A., Li V. Refractory solutions from International hazardous waste incineration experience. Proceedings of the 37th International Conference on Thermal Treatment Technologies and Hazardous Waste Combustors, IT3/HWC 2019.

[9] Villalba Weinberg A., Varona C., Chaucherie X., Goeuriot D., Poirier J. (2016). Extending refractory lifetime in rotary kilns for hazardous waste incineration. Ceram. Int..

[10] Schlegel E. (2018). Die alkalikorrosion feuerfester baustoffe, teil 1-eigenschaft von Alkalien, korrodierende stoffe, ersatzbrennstoffe. Keram. Z..

[11] Pieper C., Wirtz S., Schaefer S., Scherer V. (2021). Numerical investigation of the impact of coating layers on RDF combustion and clinker properties in rotary cement kilns. Fuel.

[12] Włodarczyk-Sielicka M., Połap D. (2019). Automatic classification using machine learning for non-conventional vessels on inland waters. Sensors.

[13] Brociek R., Chmielowska A., Słota D. (2020). Comparison of the probabilistic ant colony optimization algorithm and some iteration method in application for solving the inverse problem on model with the caputo type fractional derivative. Entropy.

[14] Mohamadi H.E., Kara N., Lagha M. (2021). Efficient algorithms for decision making and coverage deployment of connected multi-low-altitude platforms. Expert Syst. Appl..

[15] Brociek R., Słota D. (2017). Application of real ant colony optimization algorithm to solve space and time fractional heat conduction inverse problem. Inf. Technol. Control.

[16] Azadi Moghaddam M., Kolahan F. (2021). Modeling and optimization of A-GTAW process using back propagation neural network and heuristic algorithms. Int. J. Press. Vess. Piping.

